# Universal and economical experimental platform for colloidal mixing lab-on-chip in parabolic flight

**DOI:** 10.1038/s41598-025-04368-8

**Published:** 2025-06-06

**Authors:** Saran Seehanam, Hein Htet Aung, Rattanawalee Tobsri, Yossaphol Kaewkumpha, Chanud Sithipreedanant, Nachanok Kladsamniang, Witsaroot Sripumkhai, Pattaraluck Pattamang, Potiwat Ngamkajornwiwat, Wanassanan Chansataporn, Wares Chancharoen

**Affiliations:** 1 Laboratory of Artificial Intelligence and Innovation in Medicine (AIIM), Princess Srisavangavadhana Faculty of Medicine, Chulabhorn Royal Academy, 906 Kampangpetch 6 Rd., Talat Bang Khen, Lak Si, Bangkok, 10210 Thailand; 2 Thai Microelectronics Center (TMEC), National Science and Technology Development Agency (NSTDA), 51/4 Moo 1, Wang Takien District, Amphur Muang, Chachoengsao, 24000 Thailand; 3 Panyapiwat Institute of Management, 85/1 Moo 2, Chaengwattana Rd., Bang-Talat, Pakkred, Nonthaburi, 11120 Thailand; 4 Faculty of Science and Engineering, School of Civil, Aerospace, and Design Engineering, University of Bristol, Queen’s Building, University Walk, Bristol, BS8 1TR UK; 5 Faculty of Information Technology, Monash University, Wellington Rd, Clayton, VIC 3800 Australia

**Keywords:** Microgravity, Parabolic flight, Emulsification, Lab-on-chip, Fluidic device, Static mixer, Syringe pump, Deep space food system, Colloid mixing, Aerospace engineering, Electrical and electronic engineering, Mechanical engineering

## Abstract

This study presents an economical experimental platform designed to investigate colloid and emulsion mixing under parabolic flight conditions. The compact 20 kg system integrates a modular fluidic device with real-time imaging capabilities to enable the observation of fluid interactions at the millimeter scale. The platform focuses on safety, like a double containment system, while remaining accessible for quick experimental modifications. Experiments using four distinct colloids, Thailand Lunar Simulant (TLS-01A), emulsions with Span 80 (50% v/v) and Tween 80 (10% v/v), and a control without additives, enabled analysis of surface tension and particle effects on mixing behavior. Through 29 experimental trials during parabolic flight cycles, each with approximately 20 s of microgravity, the system captured fluid dynamics at 240 frames per second. The platform enables future research to observe effects of surfactant and mixer geometry in real-world scale, with potential for improvements in automation and imaging capabilities. Using a simple measure of color distribution entropy, the Span 80 sample exhibited the highest degree of mixing, with a 24.2% improvement over the microgravity control and a 19.4% increase relative to ground-based Span sample.

## Introduction

An emulsion is a heterogeneous colloidal system consisting of two immiscible liquid phases, one of which is dispersed as microscopic droplets throughout the continuous phase of the other liquid. The process of Emulsification either forms these systems or reduces droplet size in existing ones, with stability maintained by interfacial active agents such as surfactants or solid particles. Emulsions are well known because of their wide use in the food, pharmaceutical, and cosmetic industries^1^, but their capacity for encapsulation and their stability have now led to their emergence as a promising technology in other areas, such as nutrition and medication delivery media for use in deep space^2^. Deep space missions require food and medicine systems that maintain the integrity of the contents under microgravity and extend storage conditions. Emulsions excel in this context because of their capacity to encapsulate both hydrophilic and hydrophobic compounds, thereby enabling the development of compact, energy-dense formulations. For example, nano-emulsions show promise for delivering fortified beverages that can supplement astronaut nutrition while providing an extended shelf life^3^. Therefore, the use of emulsions could address the present challenges that limit long-term drug stability in space environments^4^.

**Fig. 1:**
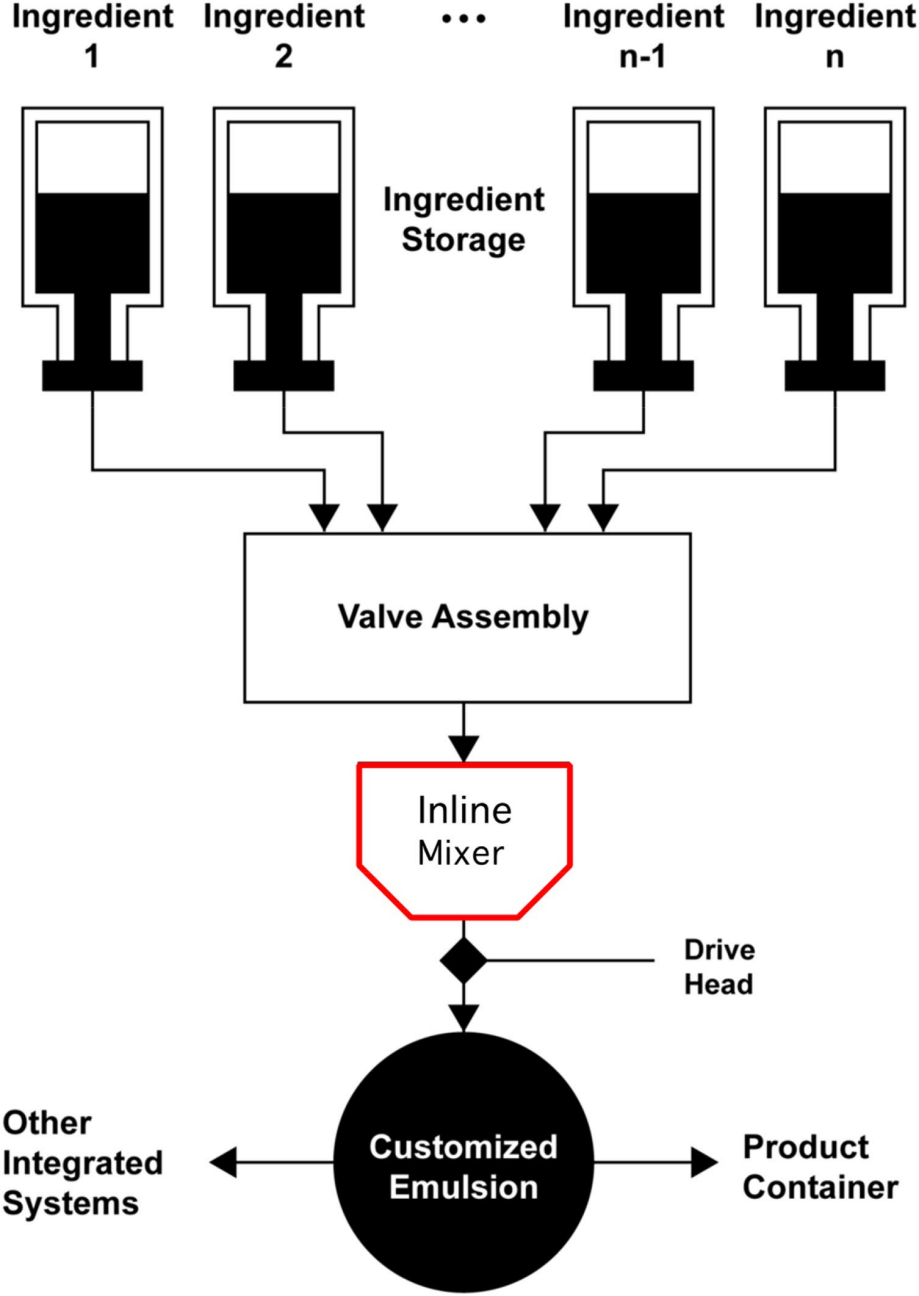
Conceptual deep-space emulsion maker.

In pharmaceutical applications, oil-in-water (O/W) and water-in-oil (W/O) emulsions facilitate the controlled release of active ingredients. Building on this foundation, advanced nano-emulsion technology has revolutionized drug delivery during space missions by utilizing submicron droplet sizes to enhance both drug solubility and bioavailability to enable precise therapeutic targeting^5^. Similarly, in astronaut food systems, space-focused emulsions provide stability against phase separation and degradation, with Pickering emulsions demonstrating superior physical stability due to their enhanced solid particle stabilization mechanism compared to traditional surfactant-based systems^6^. However, despite the versatility provided by emulsions in pharmaceutical and nutritional applications, deep space missions will still require an automated emulsification system that is capable of precise ingredient control and programmable ratios for the in-situ production of personalized therapeutic formulations and customizable food products. As shown in our conceptual model in Fig. 1, an essential component of this system will be an inline mixer capable of achieving homogeneous emulsification.

Inline mixers fall into two primary categories: dynamic mixers, which employ mechanical agitation through moving parts such as blades or impellers, and static mixers, which utilize fixed elements within a channel to blend fluids through flow-induced mixing. Static mixers, by virtue of their lack of moving components, have the benefit of design simplicity. They also offer distinct advantages for microgravity applications by reducing maintenance requirements and mechanical failure risks while accommodating diverse fluid viscosities and flow rates^7^. Overall, the optimization of static mixer geometry can significantly influence mixing efficiency and emulsion quality, with notable effects on emulsion stability^8,9^.

Another key factor that influences emulsion stability is the choice of emulsifier. Emulsifiers have an amphiphilic structure, and the combination of hydrophilic and lipophilic components reduces the surface tension between immiscible liquids^10^. The formulation parameters of emulsifiers, particularly their concentration and relative ratios, significantly influence emulsion characteristics. For example, modulating the ratio of the emulsifier Span 80 (sorbitan monooleate) to that of Tween 80 (polysorbate 80), both of which are widely utilized in pharmaceutical and food applications^11,12^, affects the hydrophilic–lipophilic balance (HLB) value, which determines emulsion type and stability^13^. Moreover, increased emulsifier concentrations typically result in reduced droplet sizes, which further enhance emulsion stability due to more effective interfacial stabilization^14^. For deep-space applications, understanding emulsification in microgravity is crucial, as density-driven forces become negligible while surface tension and rheological properties govern the process.

One way to evaluate the performance of emulsifier formulations and static mixer designs in microgravity conditions is to use parabolic flights as a testing platform. Parabolic flights offer brief periods, up to 20 s, of weightlessness^15^, thereby enabling researchers to assess critical parameters, including fluid distribution, emulsion stability, and mixing efficiency, in the absence of gravity-driven forces. Additionally, computer vision provides a noninvasive method for observing the in situ behavior of emulsion mixing under microgravity conditions^16^. In the present study, we exploited these capabilities to establish five key design criteria for developing Experiment Payload, a semi-automatic platform for noninvasive observation of emulsion behavior at the millimeter scale.

While prior microgravity emulsion research has produced valuable insights, most studies have focused on microscale phenomena and droplet formation^5,17–19^. By contrast, this work examines emulsion behavior at a more practical scale, utilizing a 4 cm × 4 cm observation chamber that better represents real-world applications in pharmaceutical and food production. Furthermore, our design incorporates inline mixing capabilities—a critical feature for deep space manufacturing that has been largely absent from previous experimental platforms. The Experiment Payload platform provides the following innovative features: (i) independent and repeatable trials that ensure consistent experimental conditions across multiple tests; (ii) precise fluid control that enables accurate manipulation of experimental materials; (iii) noninvasive monitoring that allows real-time observation without disturbing the mixing process; (iv) compliance with parabolic flight rules and regulations, thereby meeting all safety and operational requirements; and (v) ease of operation that facilitates efficient execution during limited flight windows and supports broad adoption and practical deployment.

The Design and Implementation section presents the innovations and choices made to meet the design criteria. The Experiment Payload platform is broken down into its major components, with details on their implementation. In the Operation section, key steps are outlined, from payload installation on the parabolic aircraft to its removal and subsequent data collection. The Results section presents the visual documentation of emulsion formation and mixing behavior under parabolic flight conditions and compares these to a ground-based experiment.

## Design and implementation

**Fig. 2:**
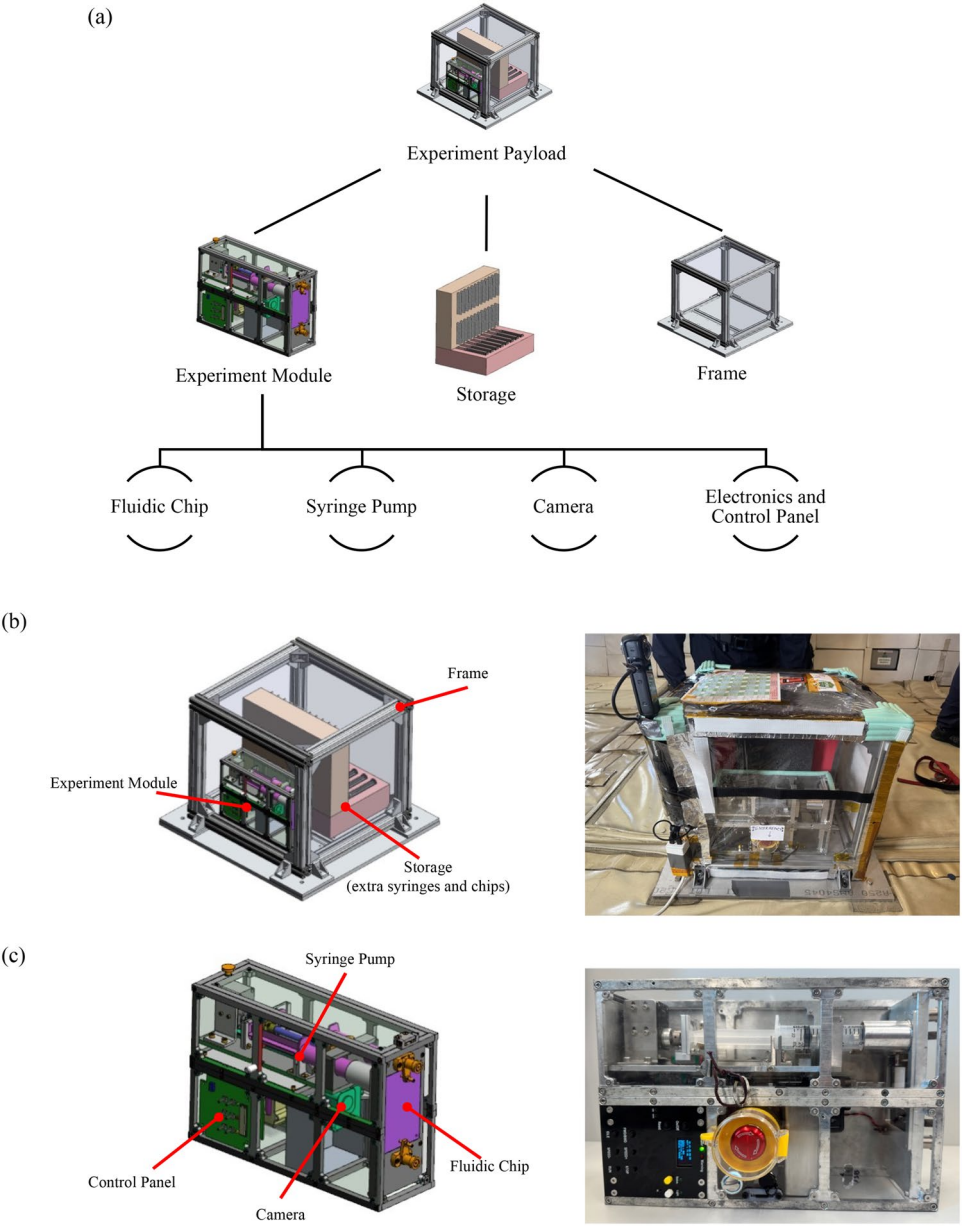
(**a**) Hierarchical organization of the design components within the experiment payload, (**b**) CAD drawing of the experiment payload with labels presented alongside the physical payload, and (**c**) CAD drawing of the experiment module, with labels presented together with the physical payload. All CAD models and 3D renderings were created by the authors using SolidWorks 2020 (Dassault Systèmes, https://www.solidworks.com/).

The experiment module is designed to extrude two different types of fluid into a fluidic device containing a static mixer and a storage chamber. Imaging with a high-speed camera through the transparent wall of the fluidic device enables in situ recording of fluid interactions. The fluidic device is designed to ensure that the camera can capture a cross-sectional view of the mixer and storage chamber. The fluid is extruded using a syringe pump designed to operate at a preset volumetric flow rate. Both the syringes and the fluidic devices can be easily replaced after use in the experiment. The integrated containment frame is constructed using aluminum profiles and plastic sheets and is designed to house the experiment module, fluid refill syringes, fluidic distribution devices, and a USB power adapter. Figure 2a illustrates the hierarchical organization of the design components. Major Components along with their functions are listed in Table 1.

**Table 1:** Major components of the design and their functions.

Major component	Function
Fluidic device(s) or fluidic chip(s)	Contains a static mixer and a storage chamber for the mixed samples
Syringe pump	Precisely dispenses the fluid
Camera	Ensures noninvasive observation of the fluid interactions
Electronics and control panel	Enables easy syringe pump operation by the user
Frame and storage	Provides a structural interface with the aircraft and an extra level of containment. Provides a safe and secure storage compartment for extra fluidic devices and syringes

The containment frame ensures that all fluid samples remain under containment during the experiment onboard the parabolic flight and that all components can be safely attached to the aircraft via a floor plate. Figure 2b shows the labeled CAD drawing of all the components placed inside the containment frame, and Fig. 2c shows the labeled CAD drawing of the experiment module, along with the respective physical prototype.

### Fluidic device

**Fig. 3:**
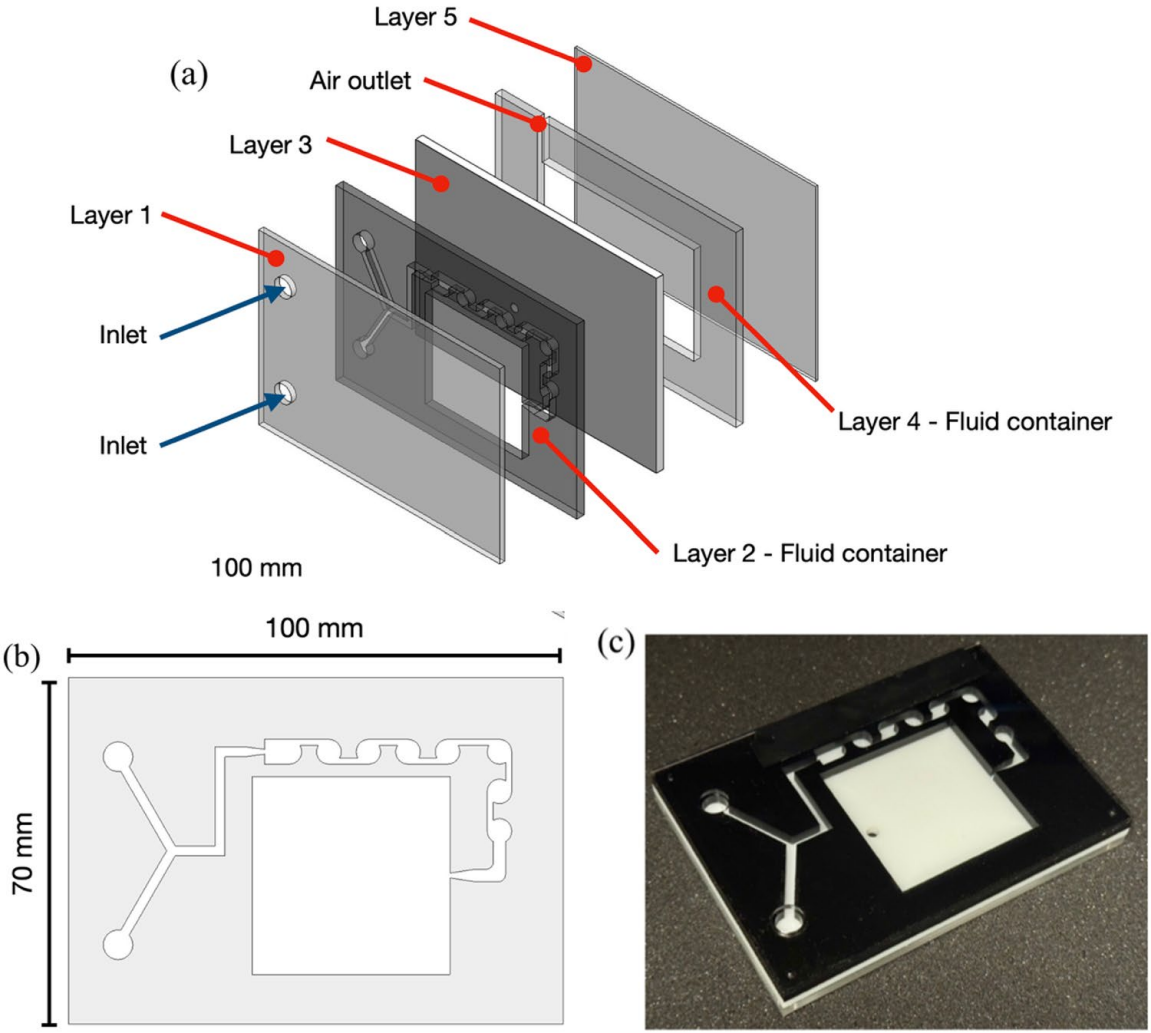
Design of the fluidic device. (**a**) Dimensions and pattern of each layer created by the authors using SolidWorks 2020 (Dassault Systèmes, https://www.solidworks.com/), (**b**) main channel pattern with dimensions, and (**c**) assembled fluidic device.

The design’s first criterion is to facilitate independent and repeatable trials through a modular approach. To achieve experimental independence, each trial must remain unaffected by previous tests, particularly by avoiding cross-contamination from residual reagents. This requirement is addressed through the implementation of a self-contained fluidic device, or “chip”, as illustrated in Fig. 3a. The chip integrates both the static mixer and the sample storage components into a single unit, thereby enabling complete isolation between trials. By replacing the entire chip after each experiment, the design ensures that subsequent trials maintain their integrity while preserving experimental repeatability. The specifications and dimensions of the fluidic mixing device were designed to fit within the experiment module, with dimensions of 70 × 100 mm. The device consists of five distinct layers, with a total thickness of 12 mm. The inlet layer (Layer 1) incorporates two inlet channels (6.5 mm diameter) designed for syringe coupling via rubber tubes (Fig. 3a). Layer 2 features the fluidic pattern (shown in detail in Fig. 3b and is optimized to enhance the mixing performance), along with a storage chamber to hold the final sample. Layer 3 serves as a wall layer, incorporating a channel 3 mm in diameter that manages overflow into the reservoir layer. The reservoir layer (Layer 4) contains excess fluid and includes a pressure-relief outlet channel sealed with parafilm to prevent leakage. The final cover layer (Layer 5) encloses the reservoir channel. The completed device assembly is shown in Fig. 3c. (See Supplementary Material—SM CAD designs, SM Fluidic Chip Fabrication).

### Syringe pump

**Fig. 4:**
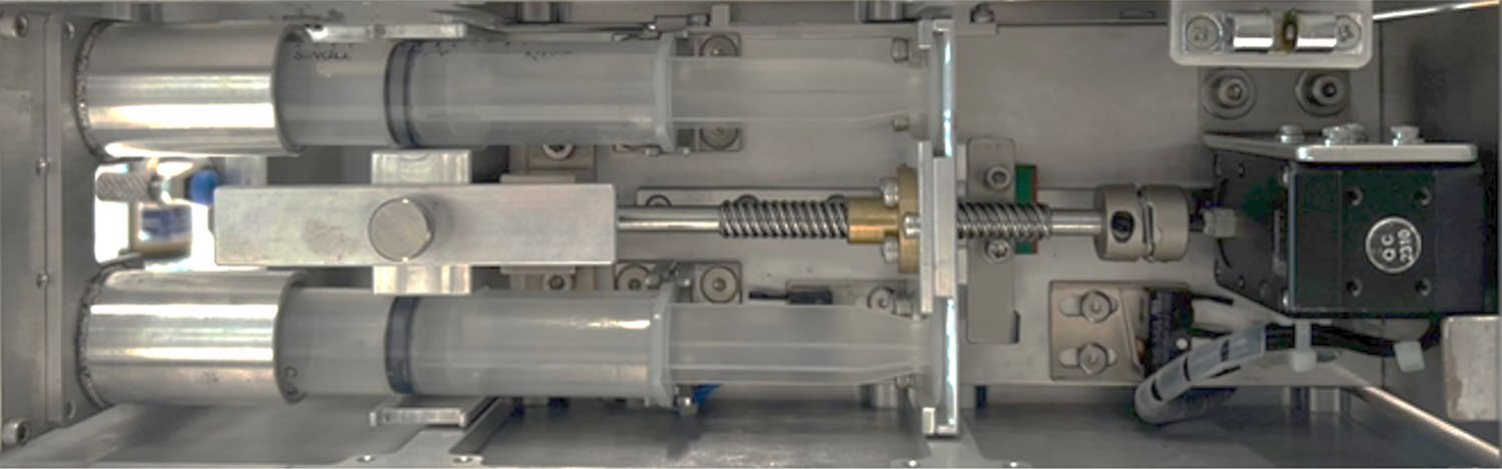
Syringe pump installed in the experiment module.

The second design criterion, precise fluid control, is achieved using a syringe pump controlled by a smart actuator. The syringe pump is designed to push the plungers of the syringe(s) at a preset velocity during each experimental trial. A Dynamixel XM430-W350-T motor (ROBOTIS, South Korea) is utilized along with a lead screw system to ensure precise fluid dispensing. The microcontroller, connected to the control panel, sets the exact velocity of the plunger, while the internal control software ensures the accurate operation of the Dynamixel motor. In this implementation, the actuator is configured so that an identical volumetric flow is achieved for all trials. However, it can also be configured to control pressure or total volume. The physical prototype of the syringe pump is shown in Fig. 4. In this design, the syringe pump is configured to hold two identical syringes and extrude the same volume from both syringes simultaneously. For other experiments, syringes with different diameters can be used to achieve varying fluid dispensing ratios.

Flow rate validation tests showed that the system maintained an average flow accuracy within ± 3.3% during ground conditions and within ± 5.0% during microgravity operations, based on measurements across multiple sample sets. These results confirm the platform’s capability to deliver stable and repeatable flow rates in both environments. Detailed measurement procedures are described in the Supplementary Material—SM Flow Rate Measurement.

### Camera

A GoPro HERO11 Black Mini (GoPro, Inc., USA) was used for noninvasive capture of fluid behavior. The durability of this camera ensures that it can withstand the mechanical stress of parabolic flight while recording data. The GoPro is securely attached to the experiment module frame using its mount holes, and the field of view is ensured to capture the whole fluidic device. Imaging the fluidic chip and fluid dynamics using the camera achieves the third design criterion of noninvasive monitoring.

### Electronics and control panel

**Fig. 5:**
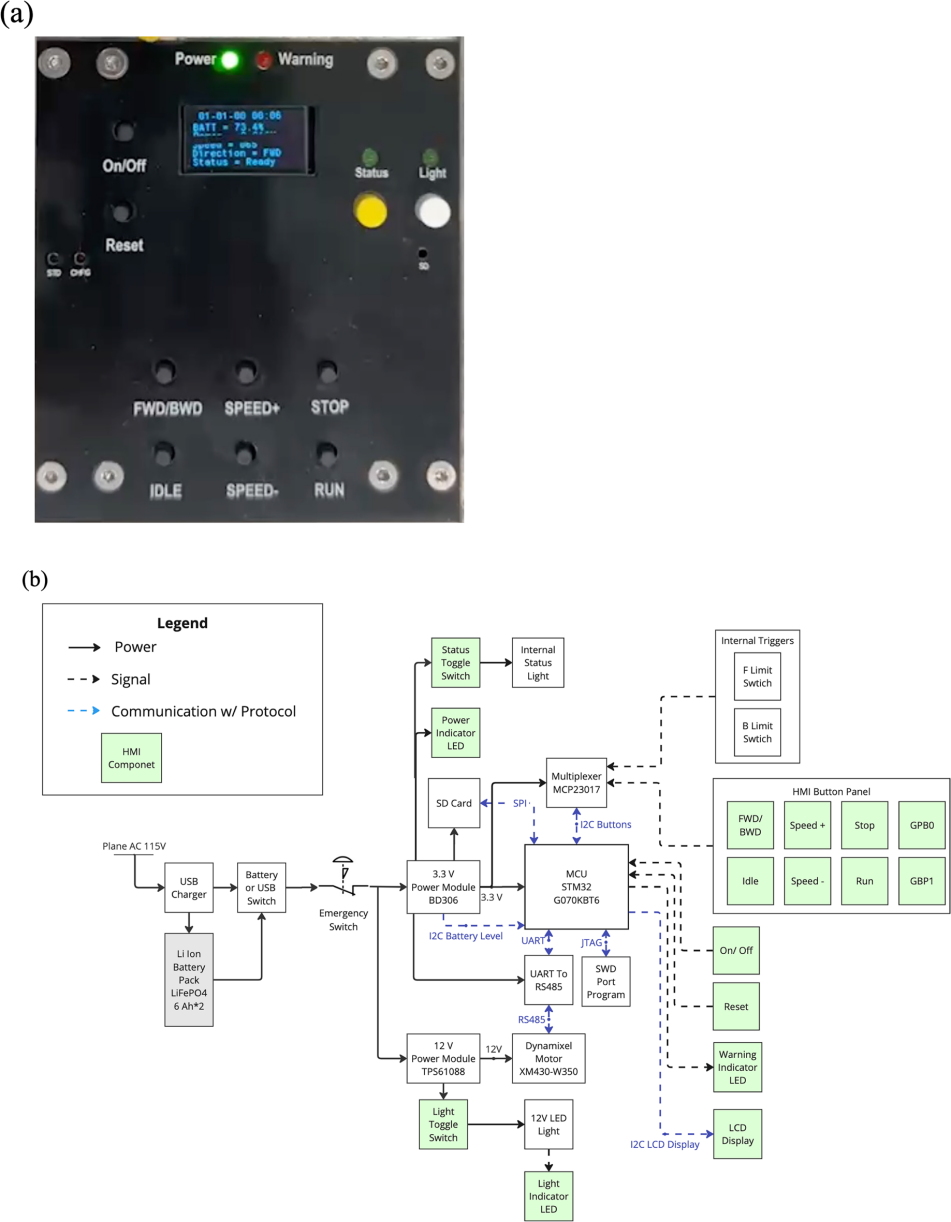
Control panel of the experiment module.

Careful design of the electronics and control panel ensures the achievement of the fourth design criterion, ease of use. The main user control panel on the Experiment Payload, shown in Fig. 5, is used to control the payload and monitor the status. The detailed operations and functions can be found in the Supplementary Material—SM Control Panel and Electronics.

For safety and compliance, an emergency stop button was integrated into the Experiment Module. This button is designed to immediately cut all internal and external power to the system in the event of operational anomalies or emergencies. To validate its performance, the emergency stop was manually triggered at various stages of experimental operation, including during syringe pump actuation and standby conditions. In all cases, the power supply to the entire Experiment Module was cut off in less than 100 ms. This rapid shutdown response ensures effective mitigation of potential risks and fulfills the safety requirements for parabolic flight operations.

### Frame and storage

The frame has dimensions of 400 × 400 × 400 mm and is covered by a transparent plastic curtain (Fig. 2a). Aluminum profiles with 20 × 20 mm cross sections are used as the main structure of the experiment’s frame. These profiles are mounted using custom-designed brackets at each corner. Polyurethane foam is chosen as the holding material for the fluidic devices and syringes with preformulated fluids. Polyurethane foam can accommodate up to 30 refill fluidic devices and 10 pairs of refill syringes. Both the main experiment module and the refill holder are installed inside the frame to ensure that none of the sample leaves the containment frame during flight. (See Supplementary Material—SM CAD Designs).

With the mechanical and structural components in place, including the containment frame, modular experiment module, and refill storage system, the Experiment Payload was fully assembled into a compact and robust platform suitable for flight deployment. To ensure that the design could support practical execution under real parabolic flight constraints, a comprehensive operation protocol was developed. This protocol outlines how the system would be set up, operated, and maintained throughout the flight cycles to maximize scientific output while maintaining safety and reliability. The following section describes the step-by-step procedures implemented during the parabolic flight campaign.

## Operation

**Fig. 6:**
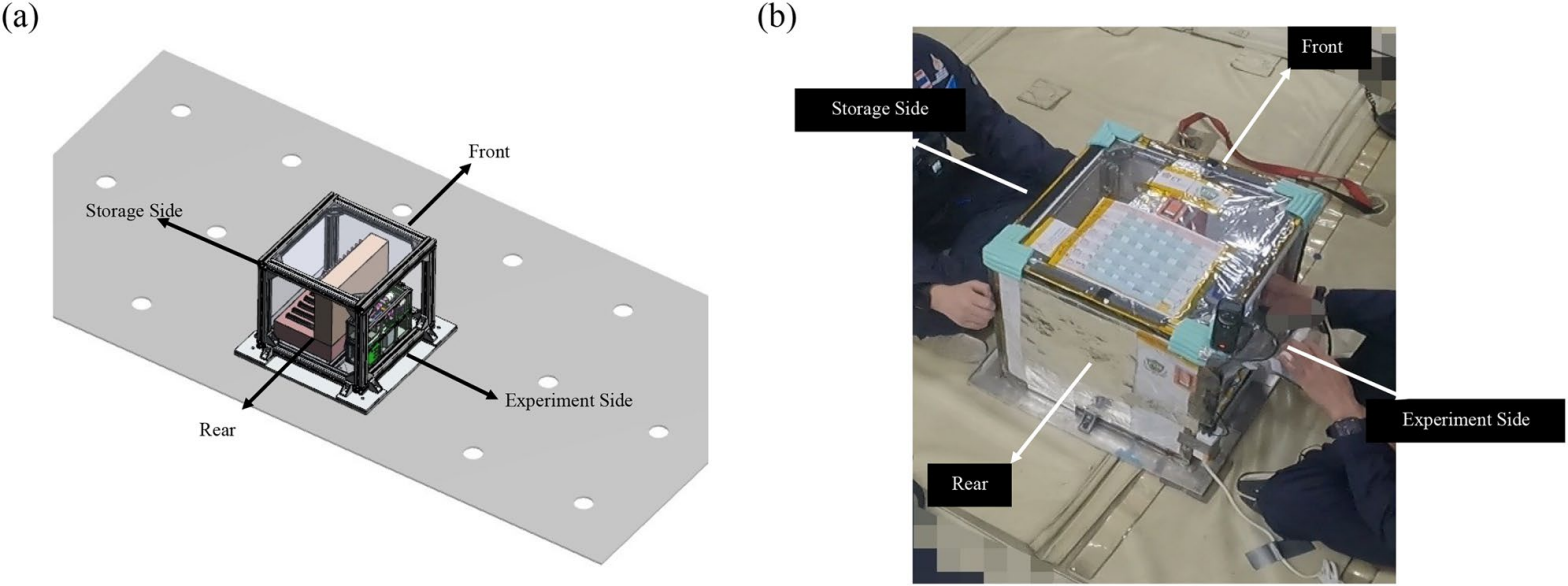
Experiment installation configuration on the aircraft. (**a**) CAD plan and layout created by the authors using SolidWorks 2020 (Dassault Systèmes, https://www.solidworks.com/), and (**b**) actual payload with experimenters.

After discussions with the parabolic flight organizer, an operation procedure was laid out to complete the maximum possible number of experiment trials (n = 29) during the parabolic flight, excluding 1 practice trial. Prior to installing the Experiment Payload on board the aircraft, a physical on-ground inspection of the experiment was conducted by the flight organizers. Once the experiment passed the inspection, its installation on the aircraft was permitted in the configuration shown in Fig. 6. One pair of syringes and one fluidic device were preinstalled in the experimental apparatus. The remaining components (10 pairs of syringes and 28 fluidic devices for reloading) were stored in the experimental storage compartment with leakage prevention tips. Liquid-absorbent materials were attached to the plastic curtain as an additional safeguard against fluid leakage. Foot straps were provided to secure the researchers during the zero-gravity operations. The experiment was designed as a two-person operation: one researcher was stationed at the side of the experiment module and the other at the side of the storage. The researcher stationed beside the experiment module was responsible for conducting the experiments, including replacing fluidic devices and syringes as needed. The second experimenter was responsible for collecting the used fluidic devices and syringes and handing the new ones to the first researcher.

A parabolic flight maneuver comprises three distinct gravitational phases. The hyper-gravity phase produces gravitational acceleration up to 1.8 G for approximately 60 s. The zero-gravity phase achieves microgravity for approximately 20 s. Following each set of 5 parabolas, a transition occurs to the steady horizontal state with 1 G acceleration, lasting 3–5 min, which serves as a rest and preparation period before the next parabolic set. The flight begins with 2 lunar gravity parabolas, producing a gravitational acceleration of 0.16 G for 20 s. In total, the flight consisted of 30 maneuvers, comprising 28 microgravity and 2 lunar-gravity conditions.

The primary materials utilized in this study were Span 80 (Sorbitan oleate, Sigma-Aldrich, USA), Tween 80 (Polysorbate 80, Sigma-Aldrich, USA), and mineral oil (Sigma-Aldrich, USA). Span 80 and Tween 80 were selected as surfactants based on their established capacity to stabilize emulsions through interfacial tension reduction between immiscible liquids^11,12^. Two distinct emulsion systems were prepared for microgravity investigation. The first system combined mineral oil premixed with Span 80 (at concentrations of 50% v/v) and DI water. The second system consisted of mineral oil premixed with Tween 80 (premixed with DI water at 10% v/v). The precise mixing ratios for all experimental materials per syringe are detailed in Table 2. For additional colloid solution experiments, Thailand Lunar Simulant (TLS-01A)^20^, which has an average grain size of 200 µm, and potato starch powder were employed to investigate particle distributions and mixing behaviors under microgravity conditions.

**Table 2:** Formulation of the sample for different experiment sets.

Experiments	DI water	Mineral oil	Span 80	Tween 80
*Emulsion set*				
Control set	100% v/v	100% v/v	–	–
SPAN set	100% v/v	50% v/v	50% v/v	–
TWEEN set	90% v/v	100% v/v	–	10% v/v
*Additional colloid set*				
Experiment	Potato starch solution		TLS-01A	
TLS-01A set	DI water 75 mL + starch 4 g		DI Water 79 mL + TLS-01A 12 g	

**Fig. 7:**
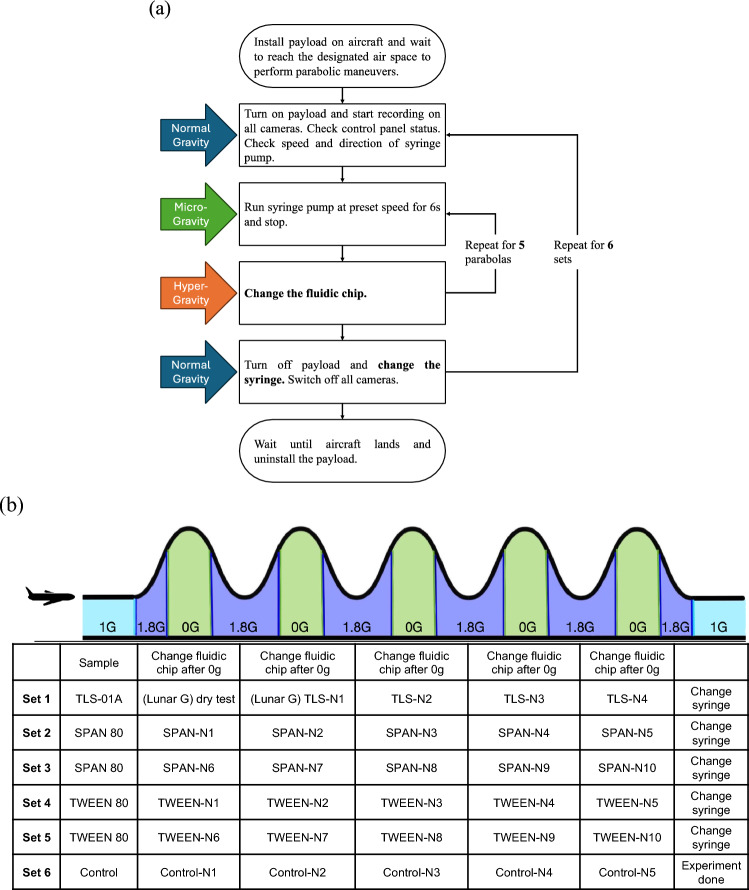
(**a**) Flow chart of operation, and (**b**) sample table for 30 parabolas.

The parabolic flight was structured by the flight organizers into six sets, each consisting of exactly five parabolas. Our experiment was organized accordingly into six Experiment Sets. The sequence began with a dry test conducted under lunar gravity conditions, followed by 29 experimental trials across the available microgravity parabolas (Fig. 7). The number of trials (n = 29) was determined by the flight schedule, which included a total of 30 parabolas. One parabola was reserved for a practice run to validate the operation procedure, and the remaining 29 parabolas were fully utilized for experimental data collection. This allocation maximized scientific output within the practical constraints of a single flight session.

The first set comprised TLS-01A and starch solution mixing experiments conducted under one lunar gravity and three zero-gravity conditions. The second and third sets focused on SPAN sample mixing, encompassing 10 experimental trials. Sets four and five were dedicated to TWEEN sample testing, which also consisted of 10 trials. The final (sixth) set served as a control group, comprising five trials without emulsifiers to establish baseline behavior. The control set enabled validation of the mixing behavior of immiscible fluids in the absence of surfactants, serving as a baseline for assessing fluid instability and observing the direct effects of microgravity on interface dynamics without the confounding influence of interfacial modifiers.

This structured approach enabled a comprehensive investigation of different fluid compositions under varying gravitational conditions. The experimental apparatus required coordinated operation between two personnel—one managing the experimental device and the other handling storage operations. The device operation followed specific protocols for different phases of the flight, as shown in Fig. 7a. Figure 7b presents a table summarizing the experimental plan across the 30 parabolic maneuvers. Each row indicates the sample set used during each parabola, allowing clear identification of the material composition tested at each flight segment. Additionally, a schematic illustration of the aircraft’s parabolic flight path is included to provide a conceptual overview of the gravitational phases encountered during the maneuvers. The illustration labels the 1.8G Hyper-gravity phase (purple), the 0G Microgravity phase (green), and the 1G Normal gravity phase (blue), indicating the relative timing and transitions between these conditions during each parabola. This contextual information aids interpretation of the experimental schedule in relation to the gravitational environment.

The experimental procedure was designed so that the most complex manipulations were performed during periods of normal gravity, as indicated by the blue arrows in Fig. 7a. During the microgravity intervals, which lasted approximately 20 s, minimal manipulation was performed to avoid disturbing the experiment. The experiment operated for 6 s to extrude the materials and then stops. The extruded materials floated in the storage chamber for the remainder of the microgravity phase. During the hyper-gravity phases, the flyers, who are taking flying onboard the parabolic flight research campaign were advised to remain still to minimize motion sickness. However, as chip changes are required during this time, a procedure was implemented to change chips while maintaining a fixed gaze at a designated point. After 5 parabolas, a set was completed, followed by 3 min of level flight under normal gravity conditions. The following are the three critical procedures that required highly coordinated movement from the two researchers:

Changing the Fluidic Device: During the hyper-gravity phase, the experimental operator unlocked and removed the fluidic device, transferring it to storage. A new device was then installed and secured using pressing pins. The used devices were sealed in zip-lock bags and stored in protective foam at the storage station. (See Supplementary Material—SM Reloading Operation).

Changing the Syringe: During the horizontal steady-state periods under normal gravity, the used syringes were transferred to storage and replaced with new sets featuring unlocked leakage tips. This process enabled continuous experimentation across multiple parabolic sets. (See Supplementary Material—SM Reloading Operation).

Emergency Protocol: In the case of operational anomalies, the experimental operator can activate an emergency switch to halt and restart the device.

leTo compare the results obtained during the parabolic flight experiment, three experimental trials of each sample set were repeated on the ground under normal gravity conditions. With the parabolic flight successfully completed and all experimental trials conducted as planned, the focus shifted to post-flight analysis and evaluation. The imaging system captured high-speed video data across multiple trials, providing a valuable dataset for assessing mixing performance under varying conditions. To extract meaningful insights, both qualitative observations and quantitative analyses were conducted. The following section presents the key results obtained from these experiments, highlighting the system’s performance in microgravity compared to ground controls.

## Results

The final prototype was easy to assemble, could be disassembled for transport, and weighed less than 20 kg. The total material cost of the major components for the entire Experiment Payload is approximately 5250 USD (See Supplementary Material—SM Material Cost). The experimental setup and procedures were pre-approved according to the documentation submitted to the parabolic flight provider. The payload successfully passed the on-site Test Readiness Review (TRR) inspection and received official approval for participation in the Fall 2024 parabolic flight campaign conducted on November 5, 2024.

Following approval, the experiment achieved all its design objectives: (i) independent and repeatable trials—the experiment was conducted across multiple trials under consistent conditions, ensuring reproducibility, and the controlled test parameters and standardized protocols confirmed the reliability of the data. (ii) Precise fluid control—the system enabled accurate manipulation of fluid flow and maintained stable conditions throughout each trial, as the precision of fluid injection and withdrawal was within the required tolerances, ensuring that mixing dynamics and interfacial interactions could be systematically studied. (iii) Noninvasive monitoring—the experiment incorporated real-time, high-resolution imaging and optical measurement techniques, allowing continuous observation without disturbing the fluid behavior, and the integration of noninvasive tracking methods ensured that the mixing process was analyzed without altering the natural progression of emulsification or droplet behavior. (iv) Compliance with flight rules and regulations—the payload was designed with strict adherence to all safety and operational guidelines for microgravity experiments, and the review of the system for structural integrity, electromagnetic compatibility, and containment measures confirmed that it met all required criteria. (v) Ease of operation—the experimental procedure was streamlined to ensure efficient operation onboard the parabolic flight, and the setup allowed for quick initialization and real-time adjustments, minimizing additional adjustments. The successful execution of the trials validated the experimental design, confirming its effectiveness in meeting all operational and scientific objectives.

**Fig. 8:**
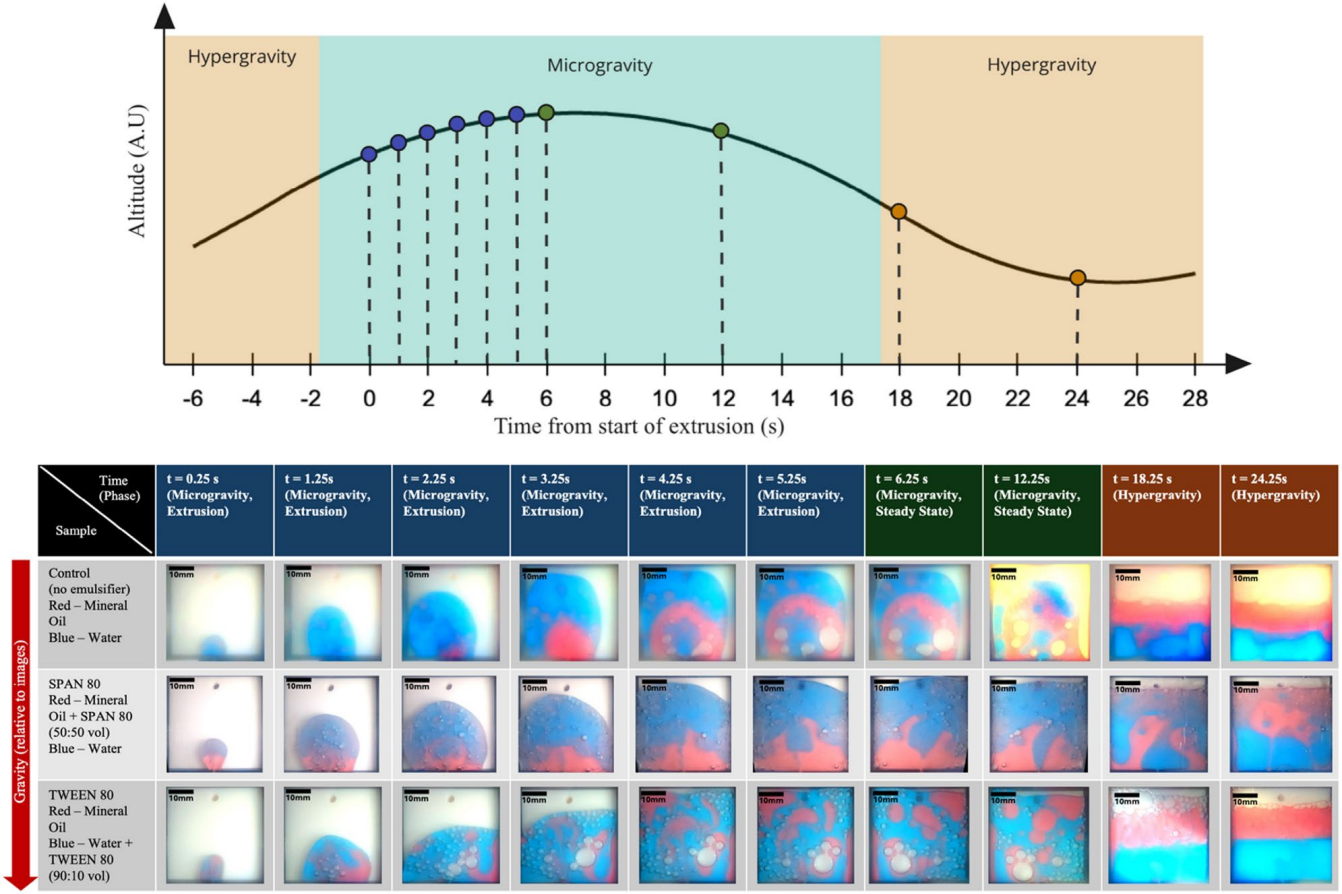
Tabulated image data from parabolic experiment trials.

**Fig. 9:**
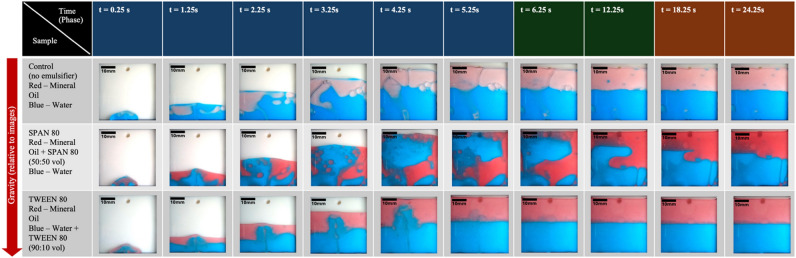
Tabulated image data from ground control trials.

To present the collected data of the experiment and evaluate the quality of the image data, images were extracted from the video recordings of three example trials chosen to represent the sample sets listed in Table 2: Control, SPAN80, and Tween80. Figure 8 presents a total of 10 images extracted from each trial. The crucial initial 6 s of extrusion are represented in six images taken at 1-s intervals, represented by blue dots on the parabolic flight profile plot. These are followed by two images capturing the steady-state phase (no extrusion) in microgravity, represented by green dots. Two images taken during hyper-gravity conditions are represented by orange dots. For this figure, t = 0 marks the initial moment when the mixture first enters the storage chamber. Note that t = 0 does not correspond to the onset of microgravity during parabolic flight. Figure 9 presents image data from the ground control trials, captured at the same 10 time points as in the microgravity trials and tabulated for the same three sample sets. Spatial calibration can be performed by referencing the known width of the storage chamber (40 mm) and measuring its corresponding pixel width in the images.

High-speed video sample from a selected trial is available for review at the “Sample Videos” folder uploaded at the Figshare repository 10.6084/m9.figshare.28405490. Due to ongoing analysis, the complete video dataset remains in processing and will be published in future work. Similarly, the results of the TLS-01A set trials are excluded from this paper, as they will be analyzed and reported in a separate publication.

To quantitatively evaluate the extent of fluid mixing in the storage chamber, Color Distribution Entropy (CDE) analysis was applied to the recorded image data^21^. Measurements were made on frames corresponding to 6.25 s after extrusion began for each trial, and a timing was selected to ensure that the extrusion process had completed and that stable post-extrusion conditions were observed. For each sample set, three independent trials were analyzed, and the resulting CDE values were averaged. CDE measures the spatial distribution of different color regions within the captured frame, providing a structured method to assess fluid dispersion. A higher CDE value indicates greater blending between the two fluid phases, reflecting a more homogeneous mixture. The results, summarized in Table 3, show that emulsions with Span 80 and Tween 80 exhibited higher CDE values under microgravity compared to their ground controls, with the baseline (control without emulsifier) displaying the lowest entropy in both conditions.

**Table 3:** Color distribution entropy after mixing.

	Microgravity (mean ± SE)	Ground control (mean ± SE)
SPAN	3.08 ± 0.14	2.58 ± 0.05
TWEEN	2.94 ± 0.03	2.30 ± 0.20
Baseline	2.48 ± 0.30	2.00 ± 0.28

## Post-experiment observation

**Fig. 10:**
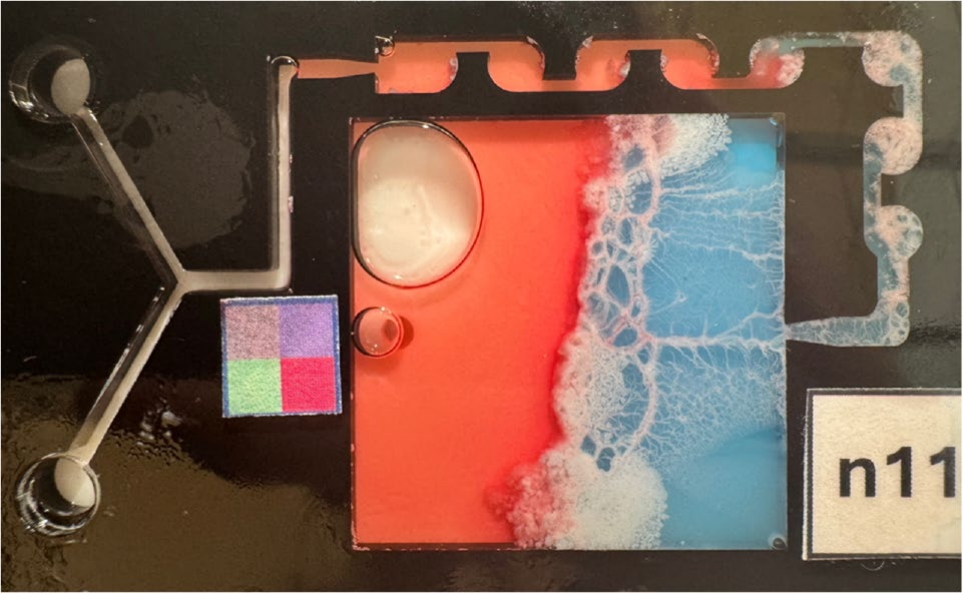
Photograph of the fluidic chip with sample captured after landing.

Upon returning to the ground, the fluidic chips were examined and photographed primarily for record-keeping purposes. However, an unexpected observation was made, as further homogenization was evident in the form of a white cream-like phase within the fluidic chip, particularly in the sample containing SPAN 80 (Fig. 10). This phenomenon could be attributed to aircraft-induced vibrations or may be the result of spontaneous emulsification^3^.

Although the scope of this study did not include investigating this effect, it presents an interesting avenue for future research. To better understand post-mixing behavior, a continuous observation camera should be installed in the future to monitor and document the state of the fluidic chips used after the experiment. With such a system, it would be possible to track the growth rate of the homogenized layer in situ, providing quantitative insights into the latent effects of mixing and phase stability.

## Discussion

In this article, the design and utilization of the Experiment Module for investigating immiscible fluid interactions at the millimetre scale have been presented, focusing partially on colloid mixing. As demonstrated by the results, the Experiment Module performed as intended and successfully recorded fluid interactions under microgravity conditions, both with and without the use of surfactants. The imaging data, which are currently under investigation for a future publication, are expected to refine analysis methodologies applicable to colloid mixing operations. Among the observations, the fluid interface exhibits the most potential for detailed analysis. In particular, the presence and type of surfactant used are significantly reflected in the evolution of the interface position, shape, and stability. Furthermore, non-invasive measurements focused on the interface are anticipated to be highly valuable for the quantification of emulsification processes at the industrial scale.

The current experimental platform successfully demonstrated its capability to investigate colloid mixing in microgravity; however, further developments are necessary for future iterations, particularly for adaptation to extended space missions, such as those on board the International Space Station (ISS). While the payload met the minimum design criteria, enhancements in automation, environmental control, and system integration are essential to improve efficiency and experimental reliability. This section discusses the key modifications needed for the current payload, the design upgrades required for an ISS deployment, and the critical considerations for developing a customizable emulsion maker for deep space. Although the payload met the design criteria set forth for this study, it remains a minimally viable product and there are potential improvements to be made. One significant challenge is the current reliance on hyper-gravity maneuvers when manually reloading the fluidic chips and syringes during experiments. Automating this process would enhance both efficiency and accuracy, thereby reducing the time and effort required for reloading and allowing for uninterrupted operation during successive parabolic flight cycles. An automated experiment presented in a previous study demonstrates the ability to manipulate and observe multiple samples without manual intervention^22^. While this system focuses solely on high-resolution imaging at the droplet scale, integrating its automated fluidic chip exchange mechanism into our fluid mixing design at a larger, real-world scale would enhance experimental efficiency and streamline operations.

Additionally, while the majority of the image data captured during the microgravity phases were of high quality, occasional interference from sunlight entering through the airplane windows introduced unwanted glare into the experimental footage. To further improve data quality, it was identified that completely blocking external light sources, including sunlight, from reaching the experimental setup would be the most effective method for ensuring consistent image quality and minimizing glare artifacts during video recording. While advanced techniques like Diffusing Wave Spectroscopy (DWS)^23^ are widely used in emulsion research, color imaging systems offer distinct advantages. DWS is an optical method that analyzes multiple light scattering events to extract information about particle dynamics in turbid media. As demonstrated in our design, color imaging enables high-speed recording while maintaining low cost and minimal space requirements. Analyzing high-resolution color images with advanced image processing techniques could uncover previously unidentified phenomena in mixing dynamics and interfacial regions.

Transitioning this platform from parabolic flights to long-term use on board the ISS presents additional design challenges^24^. One critical upgrade involves achieving a fully remote and autonomous operational mode. On the ISS, manual interventions are limited; therefore, systems must be sufficiently robust to perform unattended experiments. This would require automating the fluid loading, experimental initiation, and data logging steps. Furthermore, the system must be modular to allow easy installation and removal by astronauts without disrupting overall payload functionality. The durability of the materials used in the payload must also be reassessed following prolonged exposure to the unique environmental conditions of the ISS, including microgravity, temperature fluctuations, and potential exposure to radiation^24^. Ensuring compatibility with the ISS power and data interfaces is equally important. To systematically address these challenges and transition the payload toward ISS readiness, a step-by-step validation plan has been outlined as follows:

1. Initiate contact with ISS payload service providers to obtain detailed information on technical requirements, integration timelines, and launch opportunities.
2. Develop a prototype model with optimized mass and dimensions for ISS experimental platforms. This model will support early functionality checks and guide engineering refinements. Design considerations will follow structural safety guidance from SSP 50835^25^ and relevant constraints from the service provider.
3. Fabricate and evaluate an engineering model, implementing improvements based on prototype testing. This model will undergo environmental and structural evaluations to ensure compliance with spaceflight interface and loading conditions as described in SSP 50835^25^.
4. Conduct comprehensive electromagnetic compatibility and safety testing, including hazard analyses, containment verification, and documentation required for payload certification. EMI compliance should follow procedures outlined in MIL-STD-461^26^.
5. Proceed with payload integration and launch for in-orbit validation, following successful completion of ground-based testing and regulatory approval. In-orbit operations will focus on demonstrating autonomous performance and long-term experimental reliability under microgravity. Operations and documentation shall adhere to interface requirements defined by the payload sponsor or platform-specific Interface Requirements Documents^27^.

This structured development path ensures that the payload evolves through progressive stages of testing and regulatory review, ultimately meeting the stringent operational and safety standards required for ISS deployment.

By building on the advancements in emulsion research conducted aboard the ISS, more sophisticated emulsion manufacturing systems essential for deep-space missions can be developed in the coming decades. Unlike short-duration microgravity experiments, deep-space missions require long-term reliability, autonomous operation, and resource-efficient designs to sustain operations with minimal astronaut intervention. A key consideration is the system’s ability to consistently produce stable emulsions with precise control over droplet size, phase distribution, and mixing dynamics in the absence of gravity. Advanced microfluidic techniques could be leveraged to enable on-demand emulsion synthesis, thereby reducing fluid waste while ensuring product uniformity^28^. This would be essential for key applications, such as personalized medicine production and customized nutrient formulations for long-duration space missions. Furthermore, the use of advanced self-cleaning material is necessary to mitigate cross-contamination between different formulations to assure the quality of the product^29,30^. Finally, integrated data acquisition and real-time analysis tools must be incorporated, including high-speed imaging sensors and AI-driven analysis, to monitor and optimize droplet coalescence, aggregation, and overall emulsion stability^31^. These capabilities will enhance quality control and enable autonomous decision-making and process adjustments.

## Conclusion

The design and function of Experiment Payload successfully met its objectives, as it enabled us to use replaceable fluidic devices and syringes to conduct experiments on board a parabolic flight. Additionally, the containment frame effectively mitigated the risk of fluid leakage into the aircraft cabin. The captured high-quality video recordings provide a detailed view of fluid interactions in microgravity, facilitating further analysis. The payload design also proved to be highly portable, and the use of commercial off-the-shelf (COTS) components enhances its accessibility and ease of replication.

However, several limitations were identified. One was that the payload requires a semi-automated operation, necessitating manual interventions during hyper-gravity phases for chip reloading and level flight for syringe reloading. Only by adhering to the flight organizer’s guidelines can operators effectively manage these manual tasks during hyper-gravity conditions, which can be particularly challenging for first-time flyers and may induce motion sickness. These guidelines include maintaining a fixed head position and focusing one’s gaze on a single point throughout the hyper-gravity phase. The imaging system, while utilizing a robust commercial camera, offers opportunities for enhancement through integration of specialized scientific imaging equipment to achieve higher spatial and temporal resolution. A final area for improvement involves optimizing the payload’s thermal management for the double containment system which restrict airflow and heat exchange. To address this, a forced convection strategy is proposed, incorporating small, low-power fans placed strategically within the payload enclosure to enhance internal air circulation and promote heat dissipation. The integration of such active cooling elements would improve the thermal stability of the electronics and maintain optimal performance during extended operations.

Despite these limitations, the insights gained from this study highlight the potential of microgravity emulsion research for future applications in deep-space missions. Further advancements in this field could provide innovative solutions for sustaining human presence beyond low Earth orbit, supporting nutrition, personalized medicine, and pharmaceutical self-sufficiency in space habitats, including lunar bases and Mars missions.

## Supplementary Information

Supplementary Information.

## Data Availability

Mechanical CAD models, schematics, reloading operation videos, and sample high-speed videos are available at 10.6084/m9.figshare.28405490. Please refer to the Supplementary Materials for detailed descriptions of the available files.
