# Supplementary material for: Universal and economical experimental platform for colloidal mixing lab-on-chip in parabolic flight
Source: Sci Rep. 2025 Jun 6;15:19971. doi: 10.1038/s41598-025-04368-8 (PMC12144302; doi:10.1038/s41598-025-04368-8)
Supplement: Supplementary file 1 — Supplementary Information. [file 41598_2025_4368_MOESM1_ESM.docx]

**All files and folder mentioned in this document can be downloaded from Figshare at** **<https://doi.org/10.6084/m9.figshare.28405490>**

# SM Mechanical CAD Files

The mechanical design plays important role in the Experiment Payload as a structural support and as a frame for assembling various component together. The mechanical CAD design file can be broken down into two parts: (i) CAD file of the Experiment Module, (ii) CAD file of the payload frame and (iii) CAD file of entire Experiment Payload. The CAD file of the Experiment module contains the frame design to have the dimension of 6 cube-sat units (300mm x 200mm x 100mm); it has the fluidic chip holder, syringe pump coupling and battery pack all built in the CAD file is available as STEP file “[Experiment Module.STEP](https://figshare.com/articles/dataset/_b_Universal_and_Economical_Experimental_Platform_for_Colloidal_Mixing_Lab-on-Chip_in_Parabolic_Flight_b_/28405490?file=52317950)”. The frame of the payload provides the structure to install the main experiment module, the storage foam blocks for the extra fluidic chips and syringes, and the USB adapter. The CAD of the frame is available as “[Frame.STEP](https://figshare.com/articles/dataset/_b_Universal_and_Economical_Experimental_Platform_for_Colloidal_Mixing_Lab-on-Chip_in_Parabolic_Flight_b_/28405490?file=52317947)”. The integrated CAD of the entire payload is available as “[Experiment Payload.STEP](https://figshare.com/articles/dataset/_b_Universal_and_Economical_Experimental_Platform_for_Colloidal_Mixing_Lab-on-Chip_in_Parabolic_Flight_b_/28405490?file=52317953)”

# SM Fluidic Chip Fabrication

#
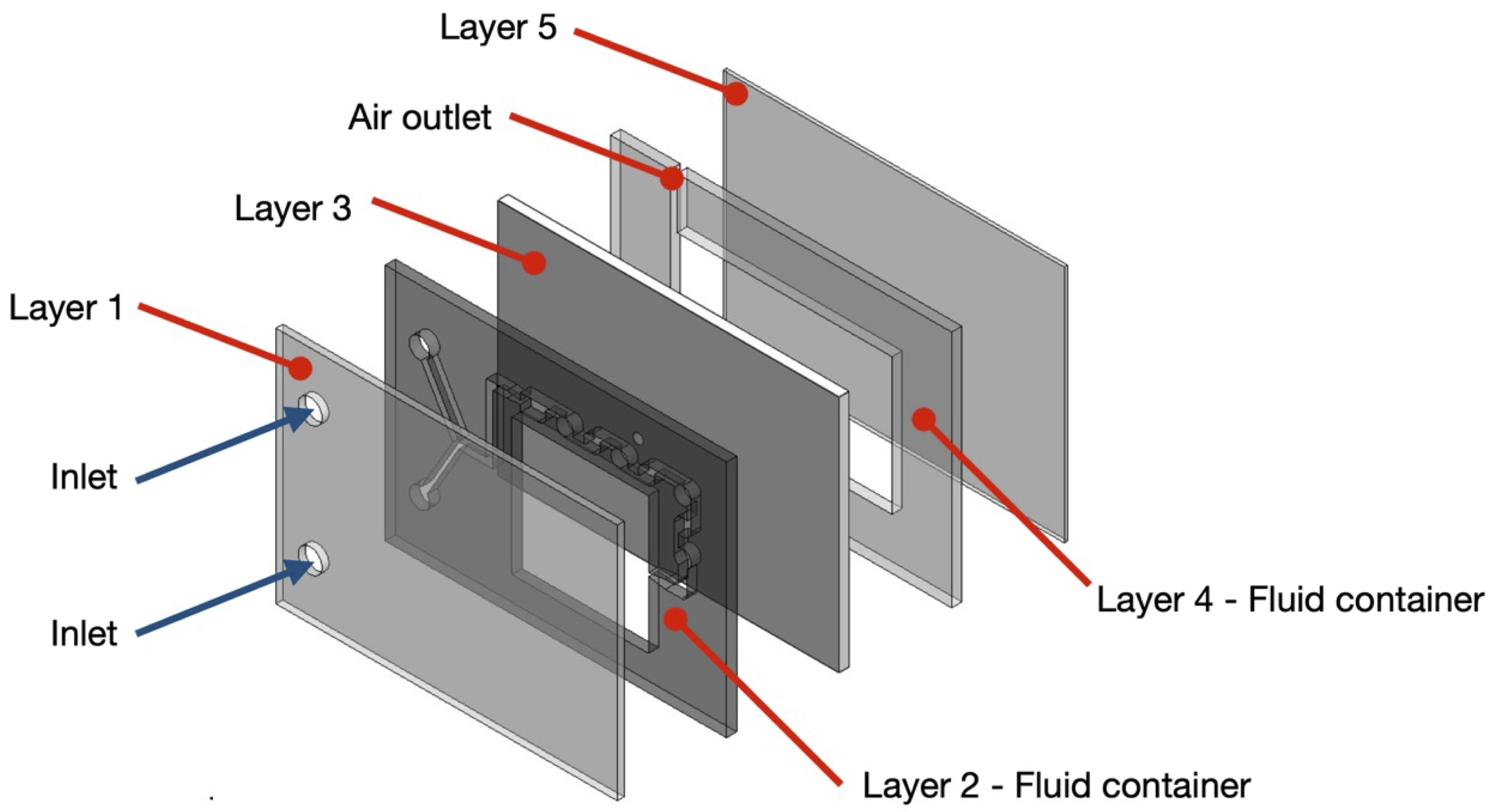


Figure S1: Design of the fluidic device’s layers created by the authors using SolidWorks 2020 (Dassault Systèmes, https://www.solidworks.com/),

The device was fabricated using a combination of transparent (Layers 1, 4, and 5) and colored acrylic sheets (black for Layer 2 and white for Layer 3) as seen in Figure S1. The use of transparent walls aids in achieving the third criterion of noninvasive monitoring, as it enables direct observation, via camera imaging, of emulsion behavior in both the mixer and storage chamber. The device was fabricated using a HYLAX HY-6040S (HYLAX, Thailand) engraving machine, with optimized parameters of 27-watt laser power and a 100 mm/min cutting rate. Following laser cutting, the acrylic components underwent ultrasonic cleaning in deionized water. The layers were assembled using pressure-sensitive adhesive film (PSA), with bonds secured by hydraulic pressing at 10 kg/cm^2^ for 120 seconds.

# SM Control Panel and Electronics

The control panel features 10 buttons, as identified in Table 2 (along with their labels and functions). The control panel also includes four indicator LEDs: a power LED for displaying the system's power status, a warning LED for signaling system alerts, a light LED that illuminates the chip during video recording, and a status LED designed for additional system monitoring but not utilized in the present study. An LCD screen is installed on the control panel to display the current status, including the set speed, set direction, battery level, regulated DC voltage, time, and readiness of the experiment module.

Table S1: Formulation of the sample for different experiment sets

| **Button No.** | **Label** | **Function** |
| --- | --- | --- |
| 1 | **Power** | Toggles the system on/off |
| 2 | **Reset** | Restarts the microcontroller |
| 3 | **Run** | Activates the syringe pump at preset direction and speed |
| 4 | **Stop** | Halts the syringe pump at its current position |
| 5 | **Idle** | Returns the syringe pump to its starting position |
| 6, 7 | **Speed + / -** | Adjusts the preset speed |
| 8 | **Fwd/Bwd** | Sets the syringe pump's direction |
| 9 | **Light** | Controls the LED that illuminates the chip during video recording |
| 10 | **Redundant** | Yellow switch is a redundant switch to add additional functions. |

The 10 buttons provide the payload operator with full control over the syringe pump operation. The input from the user control panels goes to the main microcontroller, STM32G070KB (STMicroelectronics NV, Switzerland), and sends the control signal to the Dynamixel motor via a UART to the RS485 converter. The printed circuit board (PCB) also contains a built-in power module supplied with a USB-C 5V from a power adapter that converts the aircraft's AC power to DC. The 5V is converted to 3.3V and 12V to supply various internal components. The experiment payload, designed to be portable, also has an internal LiPo4 battery, which is charged when USB power is available and used as the main power supply once the external 5V is cut off. For safety and compliance, an emergency button that can cut all internal and external power to the rest of the experiment module is also included. Figure S2(a) shows the block diagram of the electronics, showing how the various components interface and obtain their power supplies. The circuit is fabricated onto a custom PCB, and the components are soldered according to the schematic.

The schematic of the electronic circuit board and the PCB design are available in a PDF file name “[Schematic and PCB of Payload.pdf](https://figshare.com/articles/dataset/_b_Universal_and_Economical_Experimental_Platform_for_Colloidal_Mixing_Lab-on-Chip_in_Parabolic_Flight_b_/28405490?file=55054391)”. It contains the design of the power supply, main control board, motor signal transceiver and user control interface. In Figure S2(b), an overview diagram of how various components connects to the main control board via Molex ports is presented.

Figure S2:(a)Block diagram of the electronics and (b)Internal Wiring Diagram of the Experiment Module

# SM Flow Rate Measurement

The flow rate was measured by analyzing changes in pixel coverage of the fluid within the storage chamber, using high-speed video recordings. A custom image processing script was developed to facilitate this measurement process. First, users manually applied a perspective correction to the video frames to ensure accurate spatial representation of the storage chamber. The full area of the chamber was then manually selected, and a binary mask was generated to isolate the fluid region in each frame. To determine flow rate, masks were extracted at two time points: 1.00 seconds and 1.25 seconds after the start of extrusion. The change in the number of fluid-occupied pixels between these frames was used to calculate the rate of pixel increase per second.

Given that the physical dimensions of the storage chamber are 40 mm × 40 mm × 2 mm, the total volume corresponds to 3,200 mm³. By comparing this known volume with the total number of pixels covering the storage chamber, a pixel-to-volume conversion factor was established. This factor was then applied to the measured pixel change rate to calculate the volumetric flow rate in mm³/s (or mL/s). This method was applied consistently across both ground and microgravity videos to determine flow rate accuracy under each condition. And following flows where calculated. The measured average flow rates were 0.58 mL/s on the ground and 0.63 mL/s in microgravity, compared to the target rate of 0.60 mL/s.

# SM Guideline for Compliance

To fly onboard the parabolic flight organized by Zero-Gravity Corporation (FL), a safety checklist must be completed. Analyses are documented in the Payload Integration Package, which is required 60 days before the flight. These details are physically verified during the Test Readiness Review (TRR) at the airplane hangar one day before the flight.

The three key analyses for the experiment payload are:

## Structural Analysis

Conducted using finite element methods in Abaqus CAE. The model was simplified to include only the main structure, with load cases for full assembly, 1-bolt-missing, and pull-testing. Results, summarized in Table S2, show safety margins exceeding 2.0 for all cases.

Table S2: Structural Load Analysis Summary

| **Model** | **Load Case Analyzed** | **Location on Assembly** | **Maximum stress** | **Safety Margins** |
| --- | --- | --- | --- | --- |
| Full structural assembly | 9G downward | main structure | 33.82 MPa | 14.87 |
|  | 9G upward |  | 36.45 MPa | 13.80 |
|  | 9G forward |  | 16.39 MPa | 30.69 |
|  | 9G backward |  | 18.18 MPa | 27.67 |
|  | 9G lateral |  | 47.72 MPa | 10.54 |
| 1-bolt-missing | 9G downward | main structure | 116.40 MPa | 4.32 |
|  | 9G upward |  | 96.44 MPa | 5.22 |
|  | 9G forward |  | 57.46 MPa | 8.75 |
|  | 9G backward |  | 46.49 MPa | 10.82 |
|  | 9G lateral |  | 70.53 MPa | 7.13 |
| Floor plate  (Full assembly) | 9G downward | 4 mounting points attached to airplane floor | 18.38 MPa | 27.37 |
|  | 9G upward |  | 3.90 MPa | 128.97 |
|  | 9G forward |  | 4.40 MPa | 114.32 |
|  | 9G backward |  | 4.31 MPa | 116.71 |
|  | 9G lateral |  | 4.61 MPa | 109.11 |
| Floor plate  (1-bolt-missing) | 9G downward | 4 mounting points attached to airplane floor | 19.46 MPa | 25.85 |
|  | 9G upward |  | 4.18 MPa | 120.33 |
|  | 9G forward |  | 5.04 MPa | 99.80 |
|  | 9G backward |  | 4.72 MPa | 106.57 |
|  | 9G lateral |  | 5.33 MPa | 94.37 |
| Experiment payload structure | 9G downward | Attachment to main structure plate, inside the main structure | 33.19 MPa | 15.16 |
|  | 9G upward |  | 33.36 MPa | 15.08 |
|  | 9G forward |  | 42.22 MPa | 11.91 |
|  | 9G backward |  | 44.38 MPa | 11.33 |
|  | 9G lateral |  | 11.17 MPa | 45.03 |

## Electrical Analysis

Additional to the schematic provided in the above section, the load table was generated to present to total electrical power used by the payload during operation. The currents were measured in the payload by placing an ammeter in series with the power supply output and recording the various amperage consumed when different components are activated. In Table S3, the load drawn from different power supplies are summarized. Additional to load analysis emergency kill switch and procedure were documented and presented at the TRR.

Table S3: Summary of Electrical Load analysis

| Power Source Detail | Load Analysis | |
| --- | --- | --- |
| Name: Internal Battery | Motor | 265 – 500 mA |
| Voltage: 3.2 VDC | Control System | 230 – 240 mA |
| Wire Gauge: N/A | 12 V LED Light | 160 – 160 mA |
| Max Current: 3 Amps | Total Current Draw : 900 mA | |
| Power Source Detail | Load Analysis | |
| Name: USB Charger | Battery | 0 – 1 A |
| Voltage: 5 VDC | Motor | 160 – 300 mA |
| Wire Gauge: N/A | Control System | 120 – 130 mA |
|  | 12 V LED Light | 95 – 95 mA |
| Max Current: 2 Amps | Total Current Draw: 1525 mA | |
| Power Source Detail | Load Analysis | |
| Name: Extension Board | USB Charger | 0.4 – 0.6 A |
| Voltage: 115 VAC,60HZ |  |  |
| Wire Gauge: 18 AWG |  |  |
| Max Current: 20 Amps | Total Current Draw: 0.6 A | |

Although the sum of maximum current measured at individual components reaches 1.5 Amps, the battery charger is configured to divide the power between the battery and the rest of the components. The actual total current drawn from the charger does not exceed 1.2 Amps in measurement.

## Magnetic Field Analysis

Magnetic field analysis was conducted using a GM08 gauss meter (Hirst Magnetics, UK) using both the transversal and axial probe configuration with the motor running. It was measured to be less than 0.1 Gauss at 10 cm.

In addition to the required analyses, ensure that the hardware and equipment descriptions in the Payload Integration Package match the physical payload to achieve full compliance. After successfully passing the Test Readiness Review (TRR), installation onboard the aircraft must be thoroughly planned. For the payload in this study, a baseplate was rented from Zero-G Corporation to securely attach the payload to the aircraft.

## Hazardous Materials (HAZMAT) and Risk Assessment

The use of hazardous materials (HAZMAT) in parabolic flight experiments is strictly regulated by Zero-G Corporation to ensure operational safety. A thorough and precise documentation process is required, including the material's UN identification number, HAZMAT classification, and Material Safety Data Sheet (MSDS). Additionally, researchers must obtain institutional certification verifying that they are qualified to handle hazardous materials safely.

For this payload, the sample materials themselves were not classified as HAZMAT; however, the lithium-ion batteries used to power the payload and GoPro cameras were. Lithium-ion batteries pose potential fire and thermal runaway risks, necessitating additional safety documentation and compliance with UN38.3 testing requirements. This includes verification of thermal, vibration, and impact resilience, ensuring that the battery can withstand the conditions expected during parabolic flight.

To mitigate risks, all battery housings were enclosed in fire-resistant containment, and an emergency shutdown procedure was implemented in the event of overheating or electrical faults. Furthermore, the payload's battery management system (BMS) was designed to prevent overcurrent conditions that could contribute to thermal stress.

## Double Containment

As the experiment involves fluids, liquid gels, and powders, compliance with Zero-G’s double containment requirement was a critical design consideration. To achieve this, a secondary containment frame was integrated into the payload, enclosing all equipment and operations within a secure structure. This frame features absorbent curtains to capture any unintended leaks or spills, ensuring that fluids remain contained even in the event of system failure. Additionally, pre-cut and prepared rectangular liquid-absorbing pads were allocated for use by the researcher assigned to real-time spill monitoring. In the event of a minor leak, the researcher could immediately apply the absorber to contain the fluid. Used absorbent materials were then stored in a designated disposal bag attached inside the containment frame. The reloading procedure was meticulously designed to ensure that all fluid handling remains confined within the payload’s containment frame. The following section provides a detailed overview of procedures implemented to maintain safety and compliance during reloading operations.

# SM Reloading Operation

The fluidic chip requires replacement after each trial, and syringes must be changed after being used for five trials to ensure that all 29 trails are completed. However, replacing the chip and syringes poses an extra risk of fluid leaking and interfering with other experiments. A frame enclosed by plastic curtain and absorbent material is used to contain the reloading operation so that no fluid may leave the payload. The practice ground procedure with clear plastic curtain only can be available as video files in the **folder** “[SM Reloading Operations/Ground](https://figshare.com/articles/dataset/_b_Universal_and_Economical_Experimental_Platform_for_Colloidal_Mixing_Lab-on-Chip_in_Parabolic_Flight_b_/28405490?file=52317707)” These procedures were reviewed and approved by the Zero-G team to be compliant with the necessary safety and operational requirements for flight.

Zero G requires experiment to always maintain double containment of all fluid; using appropriate risk mitigation measures, a semi-automatic fluid experiment can be operated safely. The personal camera recording of the reloading a new fluidic in the actual aircraft environment during the hyper-gravity of the parabolic flight are available in the **folder** “[SM Reloading Operations/Aircraft](https://figshare.com/articles/dataset/_b_Universal_and_Economical_Experimental_Platform_for_Colloidal_Mixing_Lab-on-Chip_in_Parabolic_Flight_b_/28405490?file=52317704)”. The cameras were turned off to conserve battery at the start of each and level flight phase, so the videos of syringe changing procedure are not available.

# SM Material Cost

The material cost of the Experiment Payload was estimated based on the market prices of commercially available components, using the highest listed prices where applicable to ensure a conservative valuation. For custom-fabricated parts—including the experiment module structure, syringe pump mechanical components, and frame and storage system—the final fabrication costs were used, incorporating both material and labor expenses to provide an accurate representation of the total expenditure. All cost estimates have been rounded to the nearest 50 USD for clarity.

Table S4: Cost calculation of material in payload

| **Component** | **Cost (USD)** |
| --- | --- |
| Allylic Fluidic Chip (30 Chips) | $100 |
| Syringe Pump (including Dynamixel motor, mechanical parts and structure) | $3,000 |
| Camera (GoPro HERO11 Mini) | $300 |
| Electronics and Control Panel (including battery) | $1,500 |
| Frame and Storage (aluminum profiles, curtain and foam holder) | $250 |
| Total Estimated Cost | $5,150 |
